# Holographic Optical Tweezers That Use an Improved Gerchberg–Saxton Algorithm

**DOI:** 10.3390/mi14051014

**Published:** 2023-05-09

**Authors:** Zhehai Zhou, Guoqing Hu, Shuang Zhao, Huiyu Li, Fan Zhang

**Affiliations:** Key Laboratory of the Ministry of Education for Optoelectronic Measurement Technology and Instruments, Beijing Information Science and Technology University, Beijing 100192, China; guoqing2011@foxmail.com (G.H.); zhaos@bistu.edu.cn (S.Z.); lihuiyu@bistu.edu.cn (H.L.); zhangfan@bistu.edu.cn (F.Z.)

**Keywords:** Gerchberg–Saxton algorithm, holographic optical tweezers, spatial light modulator, optical trapping, optical manipulation

## Abstract

It is very important for holographic optical tweezers (OTs) to develop high-quality phase holograms through calculation by using some computer algorithms, and one of the most commonly used algorithms is the Gerchberg–Saxton (GS) algorithm. An improved GS algorithm is proposed in the paper to further enhance the capacities of holographic OTs, which can improve the calculation efficiencies compared with the traditional GS algorithm. The basic principle of the improved GS algorithm is first introduced, and then theoretical and experimental results are presented. A holographic OT is built by using a spatial light modulator (SLM), and the desired phase that is calculated by the improved GS algorithm is loaded onto the SLM to obtain expected optical traps. For the same sum of squares due to error *SSE* and fitting coefficient *η*, the iterative number from using the improved GS algorithm is smaller than that from using traditional GS algorithm, and the iteration speed is faster about 27%. Multi-particle trapping is first achieved, and dynamic multiple-particle rotation is further demonstrated, in which multiple changing hologram images are obtained continuously through the improved GS algorithm. The manipulation speed is faster than that from using the traditional GS algorithm. The iterative speed can be further improved if the computer capacities are further optimized.

## 1. Introduction

Optical tweezers (OTs) are a kind of optical instrument which can trap and manipulate micro-particles by using tightly focused beams, and they have been used widely in the fields of biomedicine, physics, chemistry, nanotechnology, and so on [1,2]. Holographic OTs have attracted the most attention because of their unique advantages in flexibility and applications [3], and many methods of holographic OTs have been presented in the past several years [4,5,6]. 

Spatial light modulators (SLMs) are the most commonly used optical elements in holographic OTs, which can modulate the phase and amplitude of the incident beams to further form different focused fields and then completely different trapping and manipulation capacities [7]. It is the key part for the holographic optical tweezers to load a specific phase distribution onto the incident light through an SLM, so the incident light after Fourier transform can form the desired optical field distribution in the focal region of the microscope objective. Therefore, an important research focus of the holographic optical tweezers is how to generate high-quality phase holograms through calculation.

The computer hologram algorithm that can calculate and generate multiple optical traps usually uses a direct algorithm and an iterative algorithm [8]. The direct algorithm is to directly generate holograms by dividing the SLM into corresponding regions by using a set of specified phase functions or according to the desired multiple optical traps. The iterative algorithm uses the method of continuous iteration of the calculation process to find the appropriate hologram. One of the most commonly used iterative algorithms for designing Fourier transform holograms is the Gerchberg–Saxton (GS) algorithm, which has the characteristics of simple programming, fast convergence, and strong versatility. In 2002, the GS algorithm was first introduced into the field of optical tweezers by Curtis et al. [9], and since then, GS algorithm has been widely used in many improvements and optimizations [10,11,12,13,14,15,16,17], including a weighted Gerchberg–Saxton algorithm [18,19,20]. 

An improved GS algorithm is proposed in the paper to further enhance the capacities of holographic OTs, which can improve the calculation efficiencies compared with the traditional GS algorithm. The basic theory is described in Section 2, theoretical and experimental results are presented in Section 3, and finally, the conclusions are drawn.

## 2. Basic Theory

**Figure 1:**
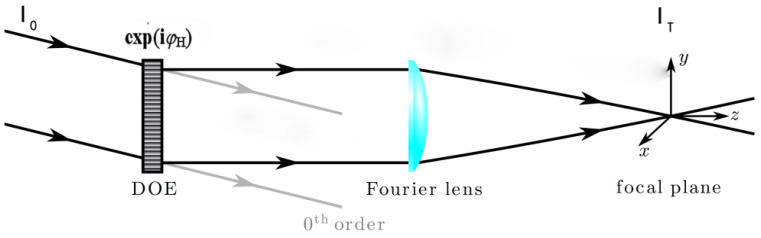
The Fourier transform model in a holographic OTs, in which DOE (diffractive optical element) usually uses SLM, the Fourier lens is an objective lens, and the focal plane is a trapping plane.

There are many structures of holographic optical tweezers, but no matter what structure it is, it can be equivalent to a Fourier transform relationship, that is, SLM is the input plane, and the focal plane (trapping plane) of the objective lens is the output plane, as shown in Figure 1. The intensity of the incident beam is *I*_0_, and the phase of the diffractive optical element (DOE or SLM) is *φ*_H_. The transmitted beam through the DOE is transformed by a Fourier lens (objective lens in OTs) into the focal plane, where its intensity is *I*_T_, so

(1) $$ℱ\left\{\sqrt{I_{0}}\exp(i\varphi_{H})\right\}\approx \sqrt{I_{T}}\exp(i\varphi_{T})$$

where ℱ{•} denotes the Fourier transform, and $\varphi_{T}$ is the phase of the transformed beam in the focal plane.

For a traditional GS algorithm, the following iteration is carried out to obtain the desired phase loaded on the SLM according to the expected focused fields.

(1) First, the initial phase is added to the predetermined incident light field as the initial phase value. Assuming that the initial phase is unknown, the value of the initial phase will usually be 0 or random.
(2) The light field on the output plane is obtained via forward Fourier transform of the initial light field on the input plane.
(3) On the output plane, keeping the phase constant, the expected optical field amplitude is used to replace the calculated optical field amplitude.
(4) Then, the light field of the input plane is obtained via inverse Fourier transform of the replaced light field on the output plane.
(5) Next, on the input plane, the amplitude of the light field is replaced by the amplitude of the desired light field while keeping the phase unchanged.
(6) Then, the forward Fourier transform is carried out again and continues to cycle until the judgment function finally converges.

**Figure 2:**
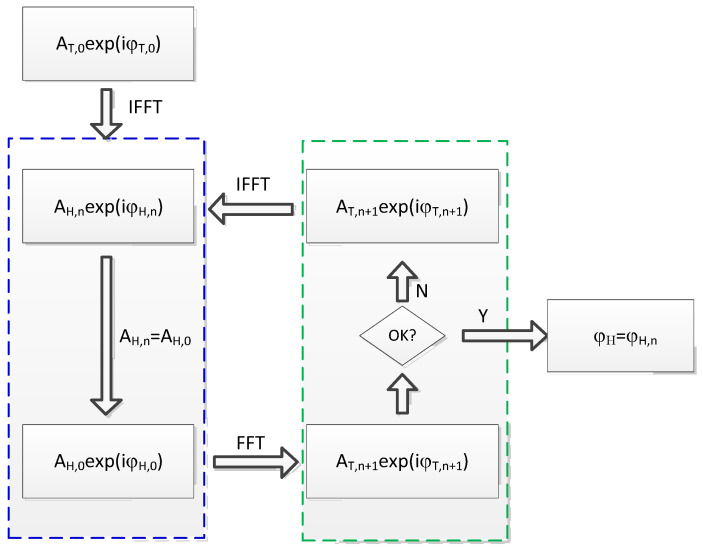
Flow chart for the improved GS algorithm, where FFT means fast Fourier transform, and IFFT means inverse FFT. The holographic plane (or input plane) and target plane (or output plane) are illustrated by the left and right dashed frame, respectively; A_H_ and A_T_ denote the amplitude of the light field on the holographic plane and target plane, respectively; and they are usually known before iteration. *φ* denotes phase, and n is the iterative number.

Now, an improved GS algorithm is presented, as shown in Figure 2, based on the traditional algorithm. Distinct from the traditional GS algorithm, at the beginning of the first iteration, a random phase factor $\varphi_{T,0}$ is chosen as the initial phase value of the expected field, and then an initial field $A_{T,0}\exp\left(i\varphi_{T,0}^{}\right)$ is obtained by setting initial amplitude factor $A_{T,0}$. Then, the inverse Fourier transform is taken, and the calculated result is chosen as the approximate value of the first iteration, so the iterative operation is performed as described above. When the iteration is performed four times, the original light field amplitude is replaced by the expected light field amplitude, that is, $A_{H,n}=A_{H,0}$; the new complex amplitude is obtained; the light field on the output plane $A_{T,n+1}\exp\left(i\varphi_{T,n+1}^{}\right)$ is obtained by Fourier transform; and the convergence judgment is made according to evaluation functions (2) and (3), as described below. If convergence is achieved, the phase distribution $\varphi_{H,n}$ is obtained as the desired phase factor; otherwise, the light field of the next iteration $A_{T,0}\exp\left(i\varphi_{T,n}^{}\right)$ is formed by setting n=n+1, $A_{T,n+1}=A_{T,0}$.

The sum of squares due to error (*SSE*) and fitting coefficient *η* are important criteria to judge the convergence of the iteration and are also the main indicators to measure the difference between the reproduction pattern and the target pattern. They are defined as follows:

(2) $$SSE=\frac{\sum {\left(A_{T,n(i)}-A_{T,0(i)}\right)}^{2}}{\sum A^{2}{}_{T,0(i)}}$$

(3) $$\eta =\frac{\sum \left(A_{Tn(i)}\cdot A_{T0\left(i\right)}\right)}{\sqrt{\sum \left(A^{2}{}_{Tn(i)}\right)\cdot \sum \left(A^{2}{}_{T0(i)}\right)}}$$

where $A_{T,n(i)}$ is the value of the *i*-th sampling point of the target plane light field amplitude, and $A_{T,0(i)}$ is the value of the *i*-th sampling point of the expected light field amplitude. The *SSE* can reflect the uniformity of the reconstructed image. When the value is closer to 0, the uniformity is better. The fitting coefficient *η* can reflect the approximation between the reconstructed image and the target image. When the value is closer to 1, the approximation is higher. In the actual iterative process, *SSE* or *η* will be used as the convergence criterion of the optimization design algorithm. When it meets the required optimal value, the iterative calculation of the algorithm can be stopped.

## 3. Theoretical and Experimental Results

In order to verify the efficiency of the improved GS algorithm, theoretical calculations are carried out. Here, a kind of reflective phase-only SLM Holoeye PLUTO NIR-011 (Holoeye company, Berlin, Germany) is used as the DOE, the resolution of which is 1920 × 1080, and pixel spacing is 8 μm. The incident beam is assumed to be a Gauss beam, the wavelength is 532 nm, and the beam waist ω is 1.5 mm.

**Figure 3:**
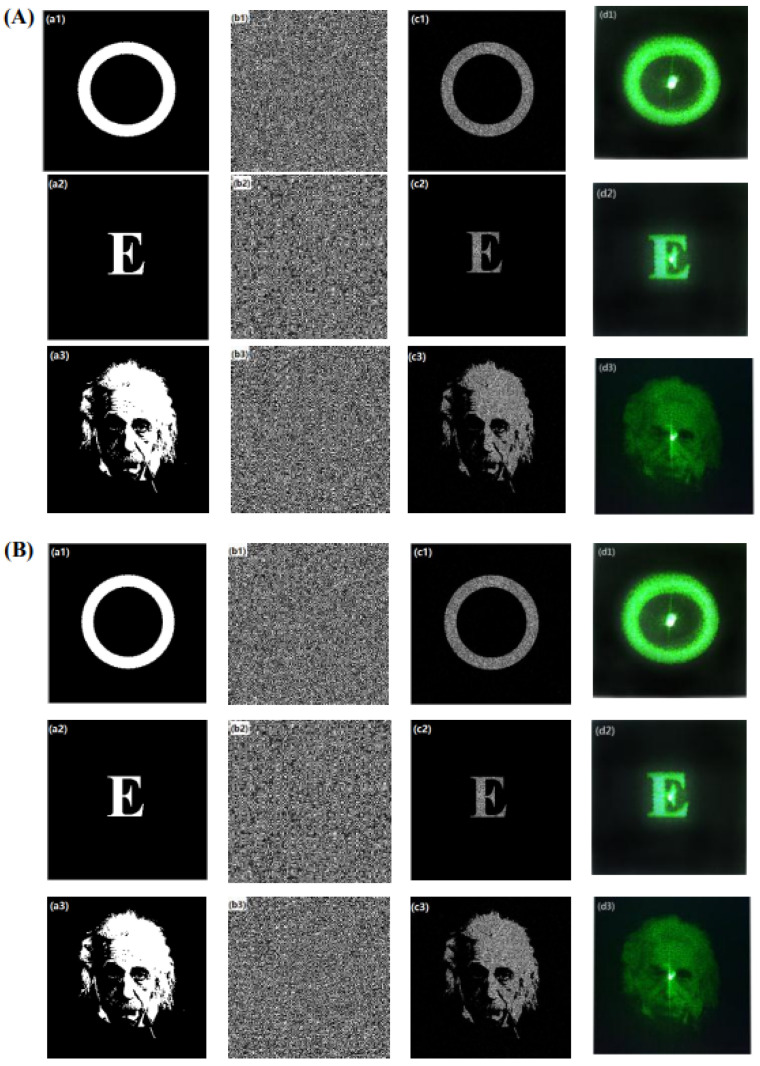
Theoretical and experimental results from using the traditional GS algorithm (**A**) and improved GS algorithm (**B**) for three different target images, namely, the desired images (**a_1_**–**a_3_**), the calculated phases (**b_1_**–**b_3_**), the reconstructed images (**c_1_**–**c_3_**), and the experimental images on the focal plane (**d_1_**–**d_3_**).

Theoretical and experimental results for three target images using the traditional GS algorithm and the improved GS algorithm, respectively, are shown in Figure 3, where the shown calculated phases (b1–b3), reconstructed images (c1–c3), and experimental images on the focal plane (d1–d3) those use the improved GS algorithm are almost same as those which use the traditional GS algorithm. However, their calculation efficiency or iterative numbers are different for both algorithms, as shown in Table 1. Moreover, when the image a1 is used as the desired image as an example, for the fitting efficient of 0.960 and the *SSE* of 0.0022, the iterative number that uses an improved GS algorithm is 22, but that number is 28 when a traditional GS algorithm is used, so the iteration speed when the improved GS algorithm is used is faster by about 27%. Furthermore, when the fitting efficient is 0.965, the *SSE* is 0.0021, and the iterative number when an improved GS algorithm is used is 72, but that number is 91 when a traditional GS algorithm is used, so the iteration speed using the improved GS algorithm is faster by about 26%. Similar results can be obtained when images a2 and a3 are used as the desired images. When image a2 is used as the desired image, the iteration speed when the improved GS algorithm is used is faster by about 26% if the fitting efficient is 0.960 and the *SSE* is 0.0022, and about 27% if the fitting efficient is 0.965 and the *SSE* is 0.0021. Similarly, for desired image a3, the iteration speeds are faster by about 26% and about 27%, respectively, for the two different conditions. The calculation time is almost proportional to the iteration numbers, so the calculation time when the improved GS algorithm is used is also faster by about 27% than that when the standard GS algorithm is used. Thus, the results show that the iteration speed is faster when the improved GS algorithm is used, which is very important for dynamic manipulation in holographic OTs.

**Figure 4:**
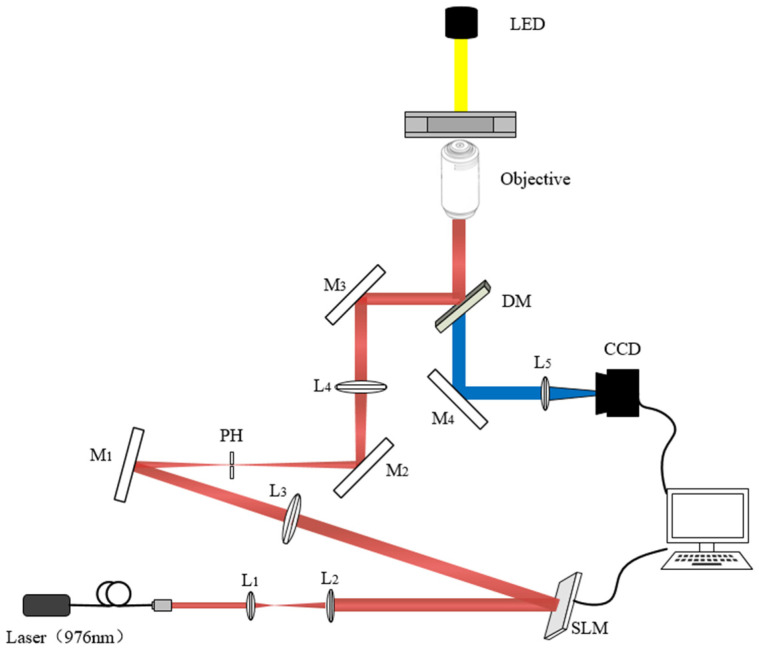
Schematic diagram of holographic optical tweezers, where L_1_, L_2_, L_3_, L_4_, and L_5_ are planoconvex lenses; M_1_, M_2_, M_3_, and M_4_ are plane mirrors; PH is the diaphragm; and DM is a dichroic mirror, reflecting the trapping light source and transmitting LED illumination light.

A holographic optical tweezer system based on the phase-only SLM is built up as shown in Figure 4, and the system can realize the trapping and dynamic manipulation of micro-particles such as cells. The lighting source is focused to the trapping area by a blue LED. The trapping light source is a 975 nm fiber Bragg grating (FBG) stabilized laser carried by the semiconductor laser controller, with an output power of 360 mw. The collimator collimates the laser into a circular spot with a diameter of about 5 mm, and the beam diameter is about 12.5 mm after passing through the beam expansion system composed of planoconvex lenses L_1_ and L_2_. The expanded beam is modulated by a phase-only liquid crystal SLM and reflected into the 4f system. The 4f system is composed of L_3_ and L_4_ flat convex lenses with a focal length of 400 mm. The L_3_ lens is placed behind the SLM with a distance equal to f, L_4_ is placed at a distance of about 2f from L_3_, and the objective lens is placed at the focal plane position of L_4_, that is, the total distance from the SLM to the objective lens is 4f, so it is called the 4f system. In order to eliminate the influence of zero-order light on the target optical trap, an aperture is placed on the confocal surface of two lenses to filter the zero-order light. After the zero-order light is filtered, the trapping beam is reflected and transmitted to the back hole of the microscopic objective through the dichroic mirror, and the optical trapping is formed on its focal plane after the objective lens. The LED light source provides the field of view. The illumination light passes through the objective lens, passes through the dichroic lens, and finally focuses the image onto the CCD through the planoconvex lens L_5_ and outputs it to the computer, which can be displayed on the display screen in real time. Types and parameters of main optical elements are listed in Table 2.

**Figure 5:**
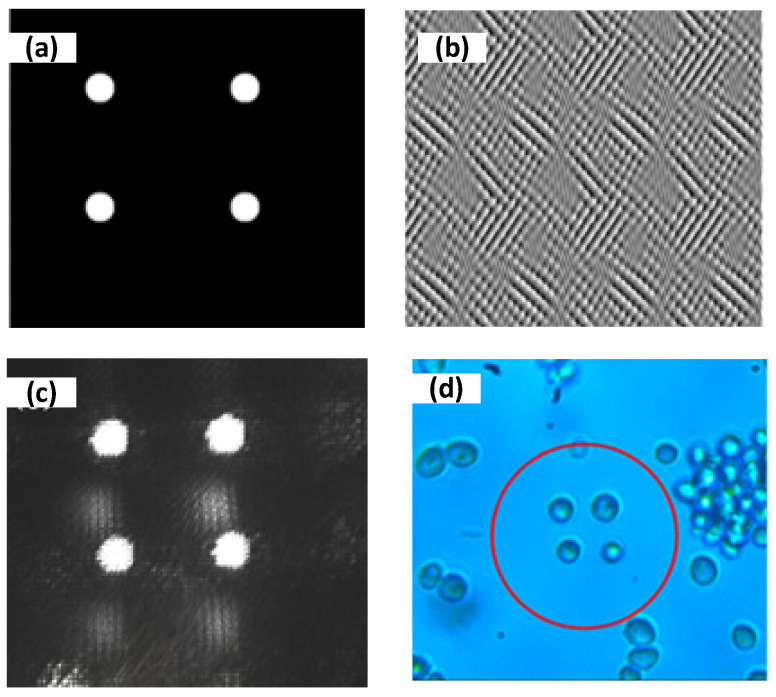
Multiple optical trapping using holographic OTs, where the number of trapped yeast cells is four; (**a**) desired optical traps, (**b**) computer-generated hologram (CGH) image loaded on the SLM, (**c**) actual optical trapping field, and (**d**) trapping of four yeast cells circled by the red circle.

First, multi-particle trapping is achieved by generating multiple optical traps. The beam modulated by SLM can obtain multiple optical traps after focusing through the objective lens. Therefore, the phase hologram used for modulating the laser beam must be obtained first. Figure 5a is the desired light field distribution. The phase hologram of the target light field is extracted by using the improved GS algorithm, and the phase hologram is loaded by using the Pattern Generator software provided by SLM. Figure 5b is the loaded phase hologram calculated by the improved GS algorithm. Figure 5c is the final actual light field distribution on the focal plane of the microscope objective. When the dissolved yeast cells are dropped into the glass culture dish, turning on the trapping light source, the yeast cells near the optical traps will be automatically sucked into the optical traps, as shown in Figure 5d, where four yeast cells are trapped stably.

**Figure 6:**
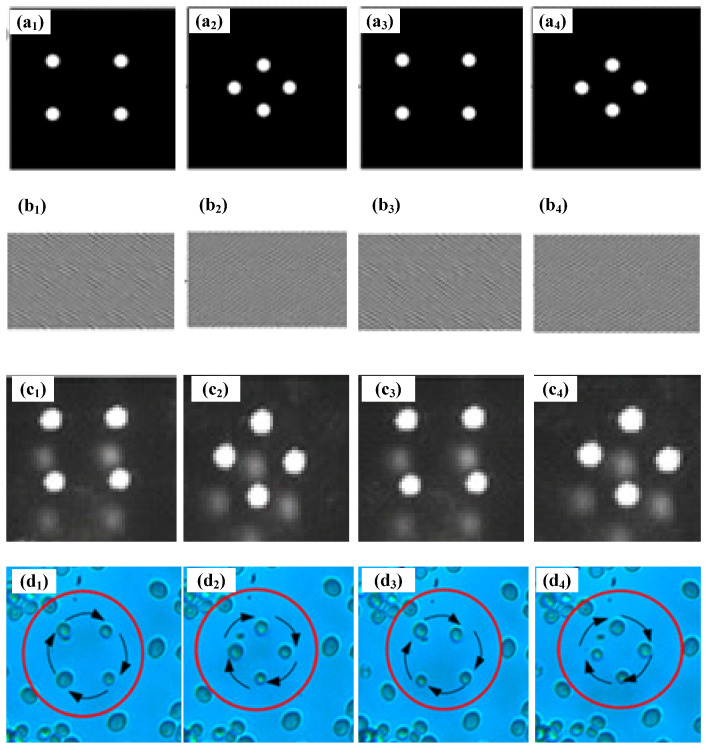
Cell rotation by using holographic OTs, where the number of manipulated yeast cells is four; (**a_1_**–**a_4_**) desired optical traps, (**b_1_**–**b_4_**) CGH images loaded on the SLM, (**c_1_**–**c_4_**) actual optical trapping fields, and (**d_1_**–**d_4_**) rotation of four yeast cells clockwise.

Next, dynamic multiple-particle manipulation is further studied, where multiple changing CGH images are obtained continuously through the improved GS algorithm and sequentially loaded onto the SLM to realize dynamically changing optical traps on the focal plane of microscope objective. Cell rotation is shown in Figure 6, in which four yeast cells are trapped and rotated by sequentially changing optical traps. Figure 6(a_1_–a_4_) show desired changing optical traps at four different stages, and Figure 6(b_1_–b_4_) present corresponding loaded CGHs onto the SLM those were calculated by the improved GS algorithm, and Figure 6(c_1_–c_4_) are the final actual light field distributions on the focal plane of microscope objective. When the dissolved yeast cells are dropped into the glass culture dish, turning on the trapping light source, the yeast cells near the optical traps will be automatically sucked into the optical traps. Then, changing CGHs are sequentially loaded onto the SLM, spatially changing optical traps are sequentially formed, and the trapped cells are rotated along the track of the optical traps. In particular, the time interval between two trapping patterns is very important for the stable manipulation of cells. If the time interval is longer, the manipulation efficiency is lower, but if the time interval is shorter, the trapped cells may escape from the determined track. Thus, an appropriate time interval is very important and is mainly up to the calculation time of CGH. The improved GS algorithm has faster iteration speed, as mentioned above, so the time interval using the improved GS algorithm is shorter compared with that using the traditional GS algorithm. In the experiment, 75 CGH pictures are generated for one cycle, and the loaded time interval of the pictures is set to 0.2 s, so 15 s is needed for one cycle and the angular velocity of rotation is 0.42 rad/s. If the traditional GS algorithm is used, the appropriate loaded time interval is about 0.25 s, so 18.75 s is needed for one cycle. It is obvious that the manipulation efficiency is increased by using the improved GS algorithm. The manipulation of rotation for cells is very useful for the study of the dynamics of cells, which can analyze the vitality and mechanical properties of cells in various living environments, thus further analyzing some influential factors and reasons to change these vitality and mechanical properties, which are usually related to some diseases or pathological changes. 

## 4. Conclusions

The dynamic manipulation of holographic OTs which use an SLM is largely affected by the iteration algorithm, the iterative speed of which will affect the manipulation efficiency of the OTs, so an improved GS algorithm is proposed and used in the holographic OTs to enhance its manipulation efficiency. As seen from the theoretical and experimental results, the improved GS algorithm has a faster convergence speed than the traditional GS algorithm, which will accelerate the manipulation of the OTs, for example, cell rotation shown in the experiments. In fact, the iterative speed can be further improved if the computer capacities are further optimized, so a shorter time for calculation can be obtained. Furthermore, the trapping capacities can be improved by optimizing the system structure, such as by adding an additional blazed grating phase to the hologram and placing a pinhole in the confocal plane of the 4f system which can block the diffraction spot of the order 0 in the middle. 

## Figures and Tables

**Table 1:** Comparison of iterative numbers for different fitting coefficients those use traditional GS algorithm (T-GS) and improved GS algorithm (I-GS).

Desired Image	*η*	T-GS		I-GS	
		*n*	*SSE*	*n*	*SSE*
a1	0.960	28	0.0022	22	0.0022
	0.965	91	0.0021	72	0.0021
a2	0.960	29	0.0022	23	0.0022
	0.965	94	0.0021	74	0.0021
a3	0.960	32	0.0022	26	0.0022
	0.965	98	0.0021	77	0.0021

**Table 2:** Types and parameters of main optical elements to build up the holographic OTs.

Types	Parameters
LED (China, Daheng company, GCI-060411)	—
Laser controller (Daheng company, GCI-0901)	—
Laser (USA, Lumentum company, S27-7402-360)	360 mW (max)
collimator (China, Daheng company, GCX-LF18PC-980)	Focal length 11.2 mm
Lenses L_1_, L_2_ (China, Daheng company, GCL-010165)	Focal length 200 mm
Lenses L_3_, L_4_ (China, Daheng company, GCL-010167)	Focal length 400 mm
SLM (Germany, Holoeye company, PLUTO-2-NIR-011)	Resolution 1920 × 1080
DM (China, Daheng company, GCC-101112)	650–1000 nm/reflection
Objective (Daheng company, GCO-2116)	60×, NA = 0.85

## Data Availability

The data presented in this study are available upon request from the corresponding author.
